# Accuracy of guided insertion of orthodontic temporary anchorage devices comparing two different 3D printed surgical guide designs: a randomized controlled trial

**DOI:** 10.1038/s41598-025-12116-1

**Published:** 2025-08-28

**Authors:** Lea Hoffmann, Tamara Katharina Kakoschke, Alexander Keller, Linus Hötzel, Uwe Baumert, Sven Otto, Andrea Wichelhaus, Hisham Sabbagh

**Affiliations:** 1 Department of Orthodontics and Dentofacial Orthopedics, Ludwig-Maxilians-Universität München (LMU), LMU University Hospital, Munich, Germany; 2 Department of Oral and Maxillofacial Surgery, Ludwig-Maxilians-Universität München (LMU), LMU University Hospital, LMU University Hospital, Munich, Germany

**Keywords:** TAD, Temporary anchorage device, Surgical guide, Mini screw, Mini-implant, Health care, Medical research

## Abstract

Temporary anchorage devices (TADs) provide maximum anchorage in orthodontic therapy. Surgical guides can be used to accurately achieve pre-planned TAD positions. The objective of this study was to assess the accuracy of guided TAD insertion in the anterior palate, comparing two 3D printed surgical guide designs. Patients were randomized in two groups. TADs were inserted using either a surgical guide with a skeletonized design or full arch design. The primary outcome was translational and rotational deviations from the virtually planned TAD positions. Secondary outcomes included the dependence of deviations on age and gender and TAD success rate after a follow-up period of three months. In total 40 patients were included in this RCT and 80 TADs were inserted. During the follow-up period, no TAD loss was observed. The skeletonized design showed significantly higher deviations between the planned TAD position and the actual TAD position in all, but two spatial dimensions (translational and rotational) compared to the full arch design. The right TAD showed higher deviations compared to the left TAD for the skeletonized design. The full arch design showed significantly higher accuracy compared to the skeletonized design. During virtual planning of TAD positions, a safety distance should be considered to account for possible deviations using 3D printed surgical guides for TAD insertion in the anterior palate.

**Supplementary Information:**

The online version contains supplementary material available at 10.1038/s41598-025-12116-1.

## Introduction

Temporary anchorage devices (TADs) are commonly used in orthodontic therapy to provide maximum anchorage for complex treatment tasks, such as maxillary expansion, distalization and mesialization of teeth, intrusion and extrusion, molar uprighting and Class II and Class III treatment^1^. The anterior palate is a favorable anatomical zone with sufficient bone availability and without tooth roots or other anatomical structures at risk^2–4^. TADs placed in the anterior palate can be used for most clinical indications and show the highest success rates compared to other intraoral regions^5^. The most common failures of TADs include insufficient implant stability and placement in an unfavorable anatomical position^5^. In addition, damage to anatomical structures, especially tooth roots, poses a risk factor for TAD placement^6^.

The use of patient-specific surgical guides was proposed to facilitate the positioning of TADs at the desired location, including insertion depth and parallelism among multiple TADs, and to avoid complications such as damage to anatomical structures^2,7^. Insertion guides can be manufactured using a conventional laboratory process or using CAD-CAM (computer-aided-design, computer-aided-manufacturing) technology by subtractive or additive manufacturing^8^. The use of digital techniques facilitates the laboratory process and allows anatomical structures such as tooth roots to be visualized using cephalometric radiographs or CBCT (cone beam computed tomography)^9,10^.

The accuracy of guided TAD insertion is decisive to prevent potential damage to anatomical structures and to ensure the best possible conditions for the insertion of orthodontic appliances. With high accuracy, the single-visit protocol can be followed to insert both, the TADs and the orthodontic appliance in the same clinical session, reducing the number of appointments required and increasing treatment efficiency^11^.

To date, most of the available studies on the accuracy of guided TAD insertion are either experimental studies, studies on human cadavers or retrospective studies, while high-quality clinical studies are lacking^7,12^. To the authors’ knowledge, no randomized clinical study investigating the accuracy of guided TAD insertion is available.

### Objectives and hypotheses

The aim of this randomized controlled trial (RCT) was to assess the accuracy of guided TAD insertion in the anterior palate, comparing two 3D printed surgical guide designs. As secondary objective, the TAD success rate was evaluated after a 3-month follow-up period. The null hypothesis was that there are no significant differences between the two surgical guide designs regarding the accuracy of TAD insertion or success rate.

## Results

### Descriptive analysis

Table 1 summarizes the patients’ characteristics. A total of 40 patients were included, of which 22 were female and 18 male. The overall mean age was 19.09 ± 9.74 years.

**Table 1:** Patients’ characteristics including mean age in years and gender distribution.

	Mean age (in years)	Gender *n* = male/female (male/female in %)
**Total**	19.09 ± 9.74	18/22 (45/55)
**Full arch design**	20.13 ± 11.9	8/12 (40/60)
**Skeletonized design**	18.06 ± 6.87	10/10 (50/50)

### Accuracy evaluation

The analyzed differences between the planned and actual TAD positions (angle between the long axis, distance at the tip, and distance at the top) are presented in Table 2.

**Table 2:** *Analysis of the planned TAD position and the actual TAD position of the two different guide designs (full arch design and skeletonized design) illustrated in total*,* for the right tads and for the left tads. A p-value of < 0.05 was considered as significant.*

	Variable	Full arch design			Skeletonized design			p-value
		Mean	SD	95% CI	Mean	SD	95% CI	
**Total**	Angle [°]	5.55	4.88	3.99 to 7.11	6.44	3.36	5.37 to 7.52	0.082
	Distance tip [mm]	1.47	1.30	1.06 to 1.89	2.00	1.01	1.68 to 2.33	**0.011**
	Distance tip in X [mm]	0.50	0.41	0.37 to 0.63	0.57	0.53	0.40 to 0.74	0.870
	Distance tip in Y [mm]	0.64	0.77	0.40 to 0.89	0.99	0.72	0.76 to 1.22	**0.008**
	Distance tip in Z [mm]	1.05	1.16	0.68 to 1.42	1.47	0.89	1.18 to 1.75	**0.008**
	Distance top [mm]	0.73	0.47	0.58 to 0.88	1.01	0.55	0.84 to 1.19	**0.021**
	Distance top in X [mm]	0.33	0.29	0.23 to 0.42	0.37	0.29	0.28 to 0.47	0.322
	Distance top in Y [mm]	0.36	0.35	0.25 to 0.47	0.61	0.50	0.45 to 0.77	**0.015**
	Distance top in Z [mm]	0.41	0.38	0.41 to 0.29	0.57	0.42	0.43 to 0.70	0.057
**Left**	Angle [°]	5.21	5.87	2.46 to 7.95	6.32	3.02	4.91 to 7.73	**0.045**
	Distance tip [mm]	1.31	1.43	0.64 to 1.98	2.15	1.02	1.67 to 2.63	**0.003**
	Distance tip in X [mm]	0.43	0.35	0.27 to 0.60	0.58	0.52	0.33 to 0.82	0.516
	Distance tip in Y [mm]	0.61	0.75	0.25 to 0.96	1.06	0.75	0.70 to 1.41	**0.020**
	Distance tip in Z [mm]	0.93	1.29	0.33 to 1.54	1.61	0.92	1.18 to 2.04	**0.003**
	Distance top [mm]	0.66	0.44	0.45 to 0.87	1.14	0.53	0.89 to 1.39	**0.003**
	Distance top in X [mm]	0.30	0.23	0.20 to 0.41	0.41	0.31	0.26 to 0.55	0.317
	Distance top in Y [mm]	0.31	0.34	0.15 to 0.47	0.64	0.50	0.40 to 0.87	**0.023**
	Distance top in Z [mm]	0.40	0.35	0.23 to 0.56	0.70	0.43	0.50 to 0.91	**0.027**
**Right**	Angle [°]	5.89	3.75	4.14 to 7.65	6.57	3.74	4.82 to 8.32	0.646
	Distance tip [mm]	1.64	1.17	1.09 to 2.19	1.85	1.01	1.38 to 2.32	0.516
	Distance tip in X [mm]	0.57	0.46	0.36 to 0.79	0.56	0.55	0.30 to 0.82	0.766
	Distance tip in Y [mm]	0.68	0.80	0.30 to 1.05	0.93	0.71	0.59 to 1.26	0.168
	Distance tip in Z [mm]	1.17	1.03	0.68 to 1.65	1.33	0.85	0.93 to 1.73	0.433
	Distance top [mm]	0.81	0.50	0.57 to 1.04	0.88	0.55	0.62 to 1.14	0.725
	Distance top in X [mm]	0.35	0.35	0.18 to 0.51	0.34	0.27	0.22 to 0.47	0.685
	Distance top in Y [mm]	0.41	0.36	0.25 to 0.58	0.58	0.51	0.34 to 0.82	0.304
	Distance top in Z [mm]	0.43	0.42	0.23 to 0.62	0.43	0.35	0.26 to 0.60	0.646

The mean angular deviation between the planned TAD position and the actual TAD position was 5.55° for the full arch design and 6.44° for the skeletonized design. The deviation at the base (distance top) was approximately twice as high as at the tip (distance tip) for both designs and both sides (left and right).

Regarding the total analysis, significant differences between the two guide designs were observed in nearly all variables except for “Angle”, “Distance tip in X”, “Distance top in X” and “Distance top in Z”. The skeletonized design showed significantly higher deviations between the planned TAD position and the actual TAD position in all those variables compared to the full arch design. When considering the left and right TADs separately, significant differences between the two guides were be observed for the left TAD: In this case as well, higher deviations between the planned TAD position and the actual TAD position in all those variables except “Distance tip in X” and “Distance top in X” compared to the full arch design. However, looking at the right TAD no significant differences are shown between the two different guide designs.

Table 3 summarizes the differences between planed TAD position and the actual TAD position of the two different guide designs (full arch design and skeletonized design) subdivided in female and male. Significant differences were observed for the full arch design for “Distance tip in X” and for the skeletonized design for “Distance top in Y”.

**Table 3:** Analysis of the planned TAD position and the actual TAD position of the two different guide designs (full arch design and skeletonized design) subdivided in female and male. A p-value of < 0.05 was considered as significant (Kruskal-Wallis-test).

	Variable	Female			Male			p-value	
		Mean	SD	95% CI	Mean	SD	95% CI		
**Total**	Angle [°]	6.00	4.84	4.53 to 7.47	6.00	3.28	4.89 to 7.10	0.523	
	Distance tip [mm]	1.74	1.36	1.33 to 2.15	1.73	0.96	1.41 to 2.06	0.595	
	Distance tip in X [mm]	0.52	0.49	0.37 to 0.67	0.55	0.45	0.40 to 0.70	0.575	
	Distance tip in Y [mm]	0.87	0.84	0.61 to 1.12	0.75	0.66	0.53 to 0.98	0.985	
	Distance tip in Z [mm]	1.24	1.17	0.89 to 1.60	1.28	0.89	0.98 to 1.58	0.622	
	Distance top [mm]	0.90	0.54	0.74 to 1.07	0.83	0.52	0.66 to 1.01	0.462	
	Distance top in X [mm]	0.34	0.27	0.26 to 0.43	0.36	0.31	0.25 to 0.46	0.938	
	Distance top in Y [mm]	0.57	0.43	0.44 to 0.70	0.38	0.44	0.23 to 0.53	0.038	
	Distance top in Z [mm]	0.48	0.42	0.35 to 0.61	0.50	0.39	0.37 to 0.63	0.692	
**Full arch design**	Angle [°]	5.66	5.78	3.22 to 8.10	5.39	3.25	3.66 to 7.12	0.639	
	Distance tip [mm]	1.47	1.60	0.80 to 2.15	1.47	0.70	1.10 to 1.85	0.194	
	Distance tip in X [mm]	0.35	0.29	0.23 to 0.47	0.74	0.46	0.49 to 0.98	**0.004**	
	Distance tip in Y [mm]	0.72	0.97	0.32 to 1.13	0.52	0.29	0.37 to 0.68	0.307	
	Distance tip in Z [mm]	1.10	1.37	0.53 to 1.68	0.97	0.80	0.55 to 1.40	0.912	
	Distance top [mm]	0.71	0.51	0.50 to 0.93	0.76	0.41	0.54 to 0.98	0.659	
	Distance top in X [mm]	0.25	0.23	0.16 to 0.35	0.43	0.35	0.24 to 0.62	0.053	
	Distance top in Y [mm]	0.42	0.40	0.25 to 0.59	0.28	0.22	0.16 to 0.39	0.639	
	Distance top in Z [mm]	0.40	0.40	0.23 to 0.57	0.43	0.37	0.23 to 0.63	0.508	
**Skele-tonized design**	Angle [°]	6.41	3.50	4.77 to 8.05	6.48	3.30	4.94 to 8.02	0.705	
	Distance tip [mm]	2.06	0.94	1.62 to 2.50	1.94	1.11	1.42 to 2.46	0.626	
	Distance tip in X [mm]	0.73	0.61	0.45 to 1.02	0.40	0.39	0.22 to 0.59	0.088	
	Distance tip in Y [mm]	1.04	0.64	0.74 to 1.34	0.94	0.81	0.56 to 1.32	0.499	
	Distance tip in Z [mm]	1.41	0.88	1.00 to 1.82	1.53	0.91	1.10 to 1.95	0.665	
	Distance top [mm]	1.13	0.49	0.90 to 1.36	0.89	0.59	0.61 to 1.17	0.083	
	Distance top in X [mm]	0.45	0.29	0.31 to 0.58	0.30	0.27	0.17 to 0.43	0.035	
	Distance top in Y [mm]	0.75	0.41	0.56 to 0.94	0.46	0.55	0.21 to 0.72	**0.009**	
	Distance top in Z [mm]	0.58	0.43	0.38 to 0.78	0.55	0.41	0.36 to 0.75	0.829	

**Fig. 1:**
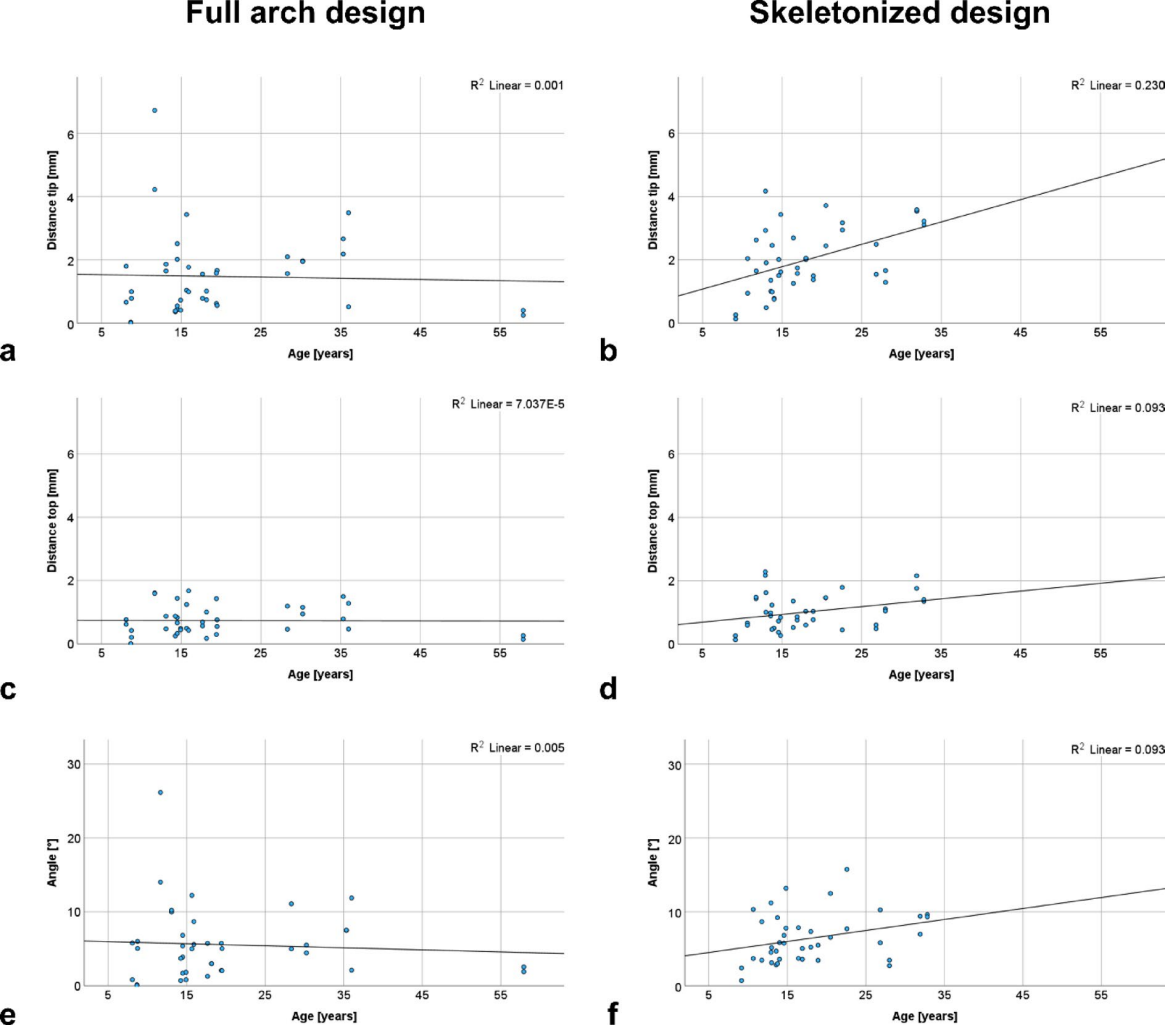
*Three-dimensional coordinate system of TADs positionings. Left TAD positioned in the 2nd quadrant*,* right TAD in the 1 st quadrant. Upper jaw image displayed in OnyxCeph version 3.2 (Image Instruments*,* Chemnitz*,* Germany*, https://onyxceph.eu*).*

Table 4 shows the analysis of the directional deviation from the planned to the actual TAD position in percent based on the predefined coordinate system shown in Fig. 1. No consistent directional deviation was observed between both guide designs. Significant differences were only observed for “Distance tip in X” for the total comparison and for the left TAD. In the skeletonized design, nearly all left TADs showed greater deviations between the planned and actual TAD positions, with two variables (“Distance top” and “Distance top in Z”) showing statistically significant differences. In the full arch design, all right TADs exhibited greater deviations; however, these differences were not statistically significant.

**Table 4:** Analysis of the orientation deviation along the X, Y and Z axis between the planned and actual TAD positions. The percentage represents the proportion of tads exhibiting a directional deviation in the negative axis direction, calculated as the cumulative count of negative vector components. A p-value of < 0.05 was considered as significant (Chi-Square-test).

	Variable	Full arch design [%]	Skeletonized design [%]	*p*-value
**Total**	Distance tip in X	37.5	70.0	**0.004**
	Distance tip in Y	75.0	75.0	1.000
	Distance tip in Z	15.0	12.5	0.745
	Distance top in X	42.5	45.0	0.822
	Distance top in Y	47.5	60.0	0.262
	Distance top in Z	45.0	25.0	0.061
**Left**	Distance tip in X	35.0	80.0	**0.004**
	Distance tip in Y	80.0	75.0	0.705
	Distance tip in Z	10.0	15.0	0.633
	Distance top in X	45.0	70.0	0.110
	Distance top in Y	50.0	60.0	0.525
	Distance top in Z	45.0	25.0	0.185
**Right**	Distance tip in X	40.0	60.0	0.206
	Distance tip in Y	70.0	75.0	0.723
	Distance tip in Z	20.0	10.0	0.376
	Distance top in X	40.0	20.0	0.168
	Distance top in Y	45.0	60.0	0.342
	Distance top in Z	40.0	25.0	0.185

The comparison of deviations between left and right TADs is shown in Table 5. For the full arch design, the left TADs demonstrated greater deviations between the planned and actual TAD positions, but these differences were not statistically significant. For the skeletonized design no consistent trend was observed, however, two variables (“Distance top” and “Distance top in Z”) showed statistically significant differences.

**Table 5:** Comparison of the two tads placed in one patient. Mean deviation of the planned TAD position to the actual TAD position of the two different guide designs (full arch design and skeletonized design). A p-value of < 0.05 was considered as significant (Wilcoxon Test).

	Variable	Left			Right			p-value
		Mean	SD	95% CI	Mean	SD	95% CI	
**Full arch design**	Angle [°]	5.21	5.87	2.46 to 7.95	5.89	3.75	4.14 to 7.65	0.391
	Distance tip [mm]	1.31	1.43	0.64 to 1.98	1.64	1.17	1.09 to 2.19	0.351
	Distance tip in X [mm]	0.43	0.35	0.27 to 0.60	0.57	0.46	0.36 to 0.79	0.108
	Distance tip in Y [mm]	0.61	0.75	0.25 to 0.96	0.68	0.80	0.30 to 1.05	0.823
	Distance tip in Z [mm]	0.93	1.29	0.33 to 1.54	1.17	1.03	0.68 to 1.65	0.232
	Distance top [mm]	0.66	0.44	0.45 to 0.87	0.81	0.50	0.57 to 1.04	0.279
	Distance top in X [mm]	0.30	0.23	0.20 to 0.41	0.35	0.35	0.18 to 0.51	0.794
	Distance top in Y [mm]	0.31	0.34	0.15 to 0.47	0.41	0.36	0.25 to 0.58	0.351
	Distance top in Z [mm]	0.40	0.35	0.23 to 0.56	0.43	0.42	0.23 to 0.62	0.526
**Skele-tonized design**	Angle [°]	6.32	3.02	4.91 to 7.73	6.57	3.74	4.82 to 8.32	0.627
	Distance tip [mm]	2.15	1.02	1.67 to 2.63	1.85	1.01	1.38 to 2.32	0.093
	Distance tip in X [mm]	0.58	0.52	0.33 to 0.82	0.56	0.55	0.30 to 0.82	0.709
	Distance tip in Y [mm]	1.06	0.75	0.70 to 1.41	0.93	0.71	0.59 to 1.26	0.526
	Distance tip in Z [mm]	1.61	0.92	1.18 to 2.04	1.33	0.85	0.93 to 1.73	0.247
	Distance top [mm]	1.14	0.53	0.89 to 1.39	0.88	0.55	0.62 to 1.14	**0.040**
	Distance top in X [mm]	0.41	0.31	0.26 to 0.55	0.34	0.27	0.22 to 0.47	0.502
	Distance top in Y [mm]	0.64	0.50	0.40 to 0.87	0.58	0.51	0.34 to 0.82	0.911
	Distance top in Z [mm]	0.70	0.43	0.50 to 0.91	0.43	0.35	0.26 to 0.60	**0.015**

**Fig. 2:**
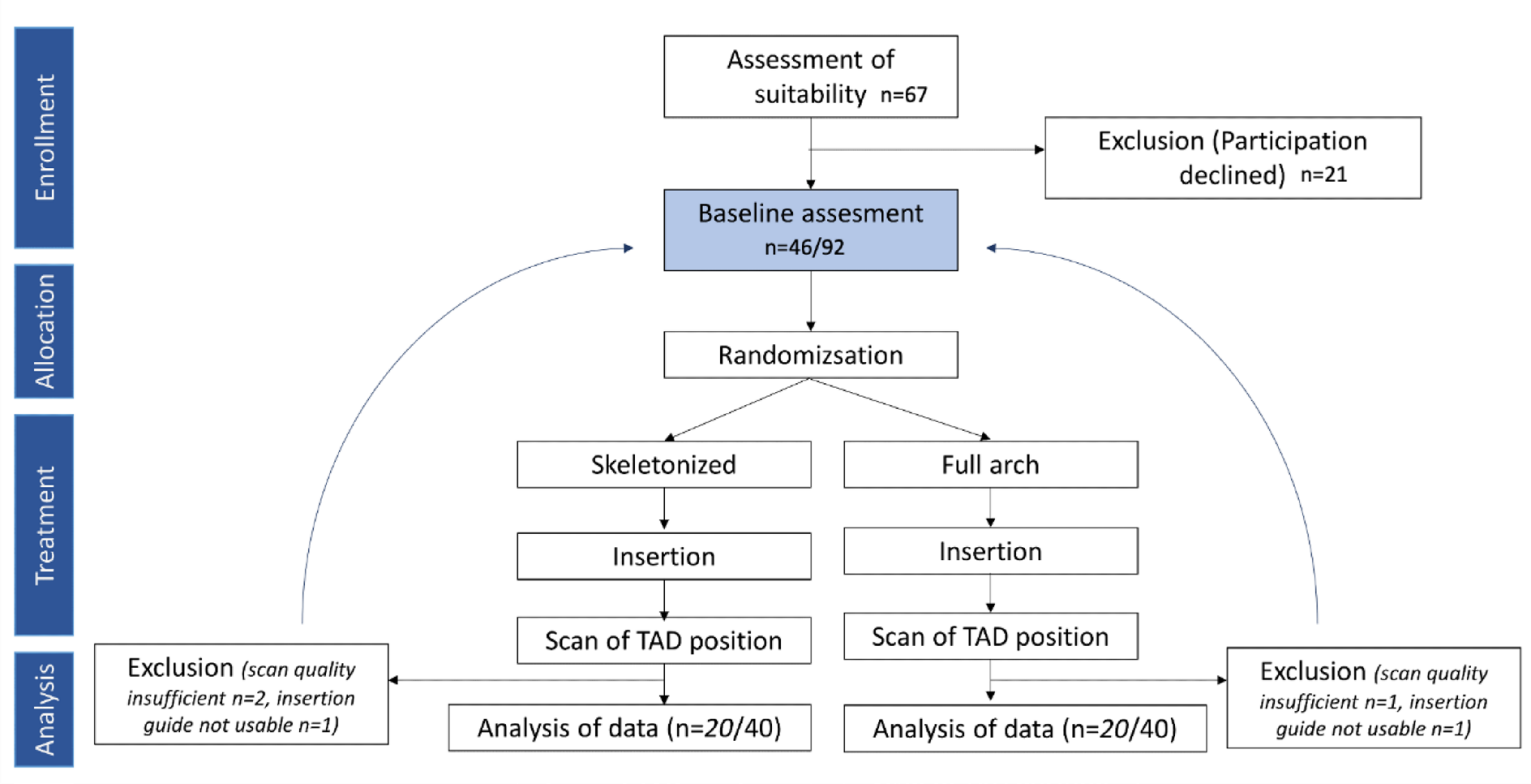
Trend analysis using regression lines of both designs. a, c, e: full arch design; b, d, f: skeletonized design. a, b: distance tip [mm]; c, d: distance top [mm]; e, f: angle [°] by age [years] of the patients.

Figure 2 presents scatter plots illustrating the trend analysis with respect to age using regression lines of key variables for the full arch design and the skeletonized design. In the full arch design, no clear relationship between age and variables was observed. In contrast, the skeletonized design showed positive trends for the three variables distance tip (Fig. 1b, R^2^ = 0.230) and distance top (Fig. 1 d, R^2^ = 0.093) and angle (Fig. 1f, R^2^ = 0.093).

### TAD success rate

After the three-month follow-up period after orthodontic loading, the success rate was 100%. No TAD showed inflammation or increased mobility, and no failures, harms or unintended effects were observed in either intervention group.

## Discussion

In the last decade, the use of TADs has substantially expanded the therapeutic spectrum in orthodontics. In addition, digital technologies enable virtual planning and precise manufacturing of customized treatment appliances. CAD-CAM surgical guides for the insertion of TADs can reduce patient discomfort, prevent damage to anatomical structures and make the procedure more precise^5,12,13^. Furthermore, guided insertion with high accuracy can increase the efficiency of treatment by reducing the number of clinical appointments^11^. However, there is limited literature on the most suitable designs of these guides to achieve accurate results. Two surgical guide design types have been proposed in experimental studies, each hypothesizing specific advantages. The full-arch design covers the soft tissues of the anterior palate and the occlusal tooth surfaces, providing a comparatively large spatial area for positioning and support^14^. In contrast, the skeletonized design is supported only on minimal occlusal surfaces, which improves the visibility of the palatal area during TAD insertion^8,14^. Based on this, the aim of this study was to compare two different CAD-CAM surgical guide designs regarding the accuracy of TAD insertion in the anterior palate in a RCT.

The mean angular deviation between the virtually planned TAD position and the scanned TAD position was 5.55° for the full arch design and 6.44° for the skeletonized design. The value ranges are consistent with the results of Pliska et al. who investigated similar CAD-CAM surgical guide designs in a human cadaver model and reported mean angular deviations of 8.23° and 12.75°, respectively^14^. However, compared to other available experimental and clinical studies the deviations are comparatively high^8,15^. It should be noted that methodological differences, not limited to the methodology of accuracy assessment, contribute to an inconsistent picture of the available data. While the study by Al-Gazzawi et al. only analyzed deviations in vertical and horizontal directions^15^the present study assessed deviations in all spatial dimensions using a highly consistent software algorithm. Mang de la Rosa et al. used a comparable methodology and reported a mean angular deviation between 0.70° and 6.03°, showing a higher deviation range which was slightly lower compared to our results^2^.

The mean difference at the tip of the TADs was 1.47 mm for the full arch design and 2.00 mm for the skeletonized design, while the difference at the top was 0.73 mm for the full arch design and 1.01 mm for the skeletonized design. Thus, deviations at the base were approximately twice as high as at the top for both designs and both sides. This finding is in line with the results from Pliska et al. who observed a similar pattern^14^. In all dimensions investigated, the skeletonized design showed a greater discrepancy between the virtually planned TAD position and the actual TAD position, which were significant in most variables (Table 2). Thus, the reduced structure and the smaller support area on the teeth and the lack of support on the oral mucosa and lower dental support may have caused the guide to shift. Both designs used required the practitioner to position the guide manually. However, the skeletonized guide possibly did not provide sufficient positional stability during TAD insertion.

In the full arch design, all right TADs exhibited greater deviations. However, no significant differences were observed. In contrast, the skeletonized design exhibited a higher deviation for the left TAD in nearly every direction, which was significant in two dimensions. The surgeon’s practice of always inserting the left TAD first, as per the clinical protocol, may explain the observed pattern. This sequence was chosen arbitrarily to standardize the workflow and minimize variability related to operator preference and not based on anatomical or clinical reasoning. Consequently, the first TAD may have caused a slight displacement of the guide, which went unnoticed and uncorrected due to the minimal dental support, leading to a greater displacement of the second TAD.

It can also be assumed that this is due to the lower positional stability of the designs. In this study, patients of all ages and genders were included and treated according to the same clinical protocol. In general, gender and age could have an influence on bone structure and density, however, a regression analysis carried out showed no correlation between accuracy, gender and age. However, since bone density was not measured, no correlation between bone density and accuracy of TAD insertion could be established. Instead, age and sex were considered as surrogate parameters due to their known association with skeletal development and bone quality. Regarding TAD stability, no differences related to age or sex were observed during the 3-month follow-up period. No TAD failures were observed, resulting in a success rate of 100% for this period. These findings align with those of a recent meta-analysis, which found no significant correlation between patient age and TAD failure rates^16^ and further support the high success rate reported in the literature for TADs in the anterior palate^5^.

Randomized clinical trials are considered the highest standard in clinical research. In this study, an automated registration method was applied using algorithms, allowing to assess deviations in all spatial dimensions without bias due to manual measurements. For the registration method, full reproducibility was shown. Therefore, the computed deviations reflect the accuracy of the CAD-CAM systems used in routine clinical procedures and protocols. However, due to the study design followed, there are some limitations to consider. The study was designed monocentric; thus, the influence of site-specific factors could not be determined. All TADs were inserted by one experienced and trained surgeon, therefore it was not possible to determine the dependence on the practitioner in terms of inter-operator variability.

Further clinical and technical aspects were not considered: no predrilling was performed, as the implants used are self-drilling and self-tapping according to the manufacturer’s specifications. Only one type, length and diameter of TADs was investigated, which limits the generalizability of the results. All TADs were inserted into the anterior palate, therefore no conclusions can be drawn on the accuracy in other insertion regions, such as interradicular, interforaminal and zygomatic crest.

In summary, the present results cannot be generalized to the overall accuracy of guided TAD insertion with CAD-CAM manufactured guides.

Further studies are needed to expand the available data on the most appropriate guide designs, manufacturing processes and clinical protocols to achieve high accuracy in various clinical scenarios. Additionally, a larger sample size should be considered to reliably assess significance in all dimensions and identify true differences between different designs. In this context, future research should also investigate the relationship between TAD accuracy and appliance fit, particularly with regard to different treatment protocols such as single-visit versus two-visit workflows.

The present results indicate that CAD-CAM surgical guides, which must be positioned manually, may have a higher clinical inaccuracy than could be expected from the limited available literature. To avoid damage in all included cases - or in the case with the largest observed deviation - a safety margin of 3.0 mm between the implant apex and the neighboring tooth structures would have been required. These results emphasize the variability in clinical accuracy when using CAD-CAM surgical guides and highlight the need for careful consideration of safety margins in virtual planning for TAD placement in the anterior palate. Safety margins should be considered to account for scanning and manufacturing tolerances as well as the potential impact of clinical circumstances. However, the mean deviation in the apex region was significantly lower and ranged between 1.47 and 2.00 mm. Therefore, maintaining a minimum safety distance of 2.0 mm appears justified based on the present data and is consistent with recommendations from previous studies^17^. In cases where root proximity is unclear or borderline, the use of 3D imaging such as cone-beam-computed tomography (CBCT) may be considered to verify spatial relationships and confirm safety margins.

## Methods

### **Trial design and changes after trial commencement**

The study was designed as a prospective, single-center, two-arm, parallel RCT with a 1:1 allocation ratio. The trial was registered at the German Clinical Trials Register (DRKS-ID 00028969) on 16/05/2022. No changes were made after initial commencement.

### Sample size calculation

At time of study initiation (2021), no comparable data were available to guide the sample size calculation. Therefore, the sample size was determined based on a significance level (α) of 0.05 and a power (1-β) of 0.95. The allocation ratio was set to 1:1 between the groups, and a two-tailed t-test was chosen to detect a mean difference between the groups with an expected effect size (Cohen’s *d*) of 0.8, resulting in a calculated sample size of 40 implants or 20 patients per group. Calculation was performed using G*Power software (version 3.1.9.6 for macOS)^18^.

### **Participants**,** eligibility criteria and settings**

**Fig. 3:**
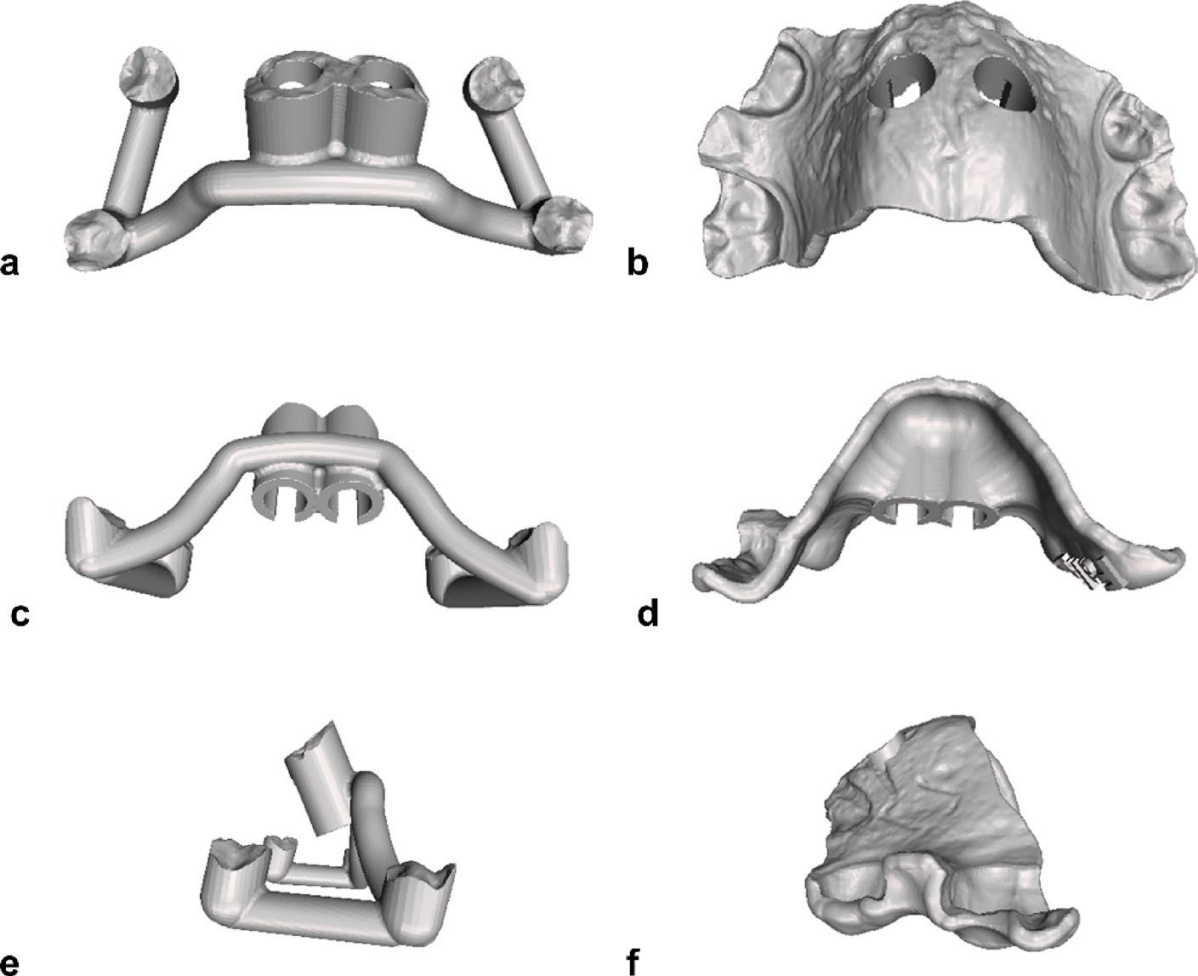
The study aimed to include 40 TADs per group (a total of 80 TADs). Recruitment was conducted based on the inclusion and exclusion criteria. If a patient initially included in the study was later excluded (e.g., due to refusal to participate or insufficient scan quality), the respective lot (if already conducted) would be returned, and additional patients were recruited. Thus, the study conclusively included the planned 20 patients (40 TADs) per group.

Patients from the Department for Orthodontics and Dentofacial Orthopedics, LMU University Hospital, were asked to participate. Patient enrollment started on May, 8th 2022 and finished on April, 1 st 2024. The randomized controlled trial (RCT) concluded following the completion of the three-month follow-up period for the final enrolled patient (Fig. 3). The study aimed to include 40 TADs per group (a total of 80 TADs). Recruitment was conducted based on the inclusion and exclusion criteria. If a patient initially included in the study was later excluded (e.g., due to refusal to participate or insufficient scan quality), the respective lot (if already conducted) would be returned, and additional patients were recruited. In total, 60 patients (120 TADs) were recruited for suitability, of which 18 (36 TADs) were excluded. Thus, the study conclusively included the planned 20 patients (40 TADs) per group.

The following inclusion criteria were applied:

1. Patients of all age groups and genders.
2. Orthodontic indication for skeletal anchorage with TADs in the anterior palate.
3. Availability of complete diagnostic records.

The following exclusion criteria were applied:

1. Oligodontia (lack of 6 or more teeth).
2. Insufficient bone or presence of teeth in the area of planned insertion (anterior palate).
3. Allergies to anesthetics.
4. Oral pathologies (active caries, parodontitis).
5. Systemic bone disease.

The study was approved by the Ethics Committee of the LMU Faculty of Medicine (13 April 2022, Ref. 22–0158). All methods were performed in accordance with the Declaration of Helsinki and relevant guidelines and regulations. Patients (and parents or guardian in the case of underage patients) received oral and written information about the study. Upon agreement to participate, informed consent was obtained.

### Interventions

#### Virtual planning of TAD positions

For the virtual planning of TAD positions and design of the insertion guides, an intraoral scan of the patients’ upper jaws were taken (Trios 3, 3shape A/S, Copenhagen, Denmark). The scans were processed using orthodontic imaging software (OnyxCeph^3TM^, Image Instruments, Chemnitz, Germany). The positions of the TADs were planned in the TADmatch module of the software, and two TADs of the dimensions 2.3 × 9.0 mm were virtually placed (BENEfit^®^, PSM Medical Solutions GmbH, Gunningen, Germany). In each individual case, TADs were planned paramedian, in the T-zone of the anterior palate according to literature recommendations^3^. Cephalometric radiographs were used during virtual planning to assess the morphology of the anterior palate and the position and inclination of the anterior incisors. No additional radiographs, such as CBCT, were used for the placement of the TADs, as the insertion was carried out in the T-zone^4^.

#### Preparation of insertion guides

**Fig. 4:**
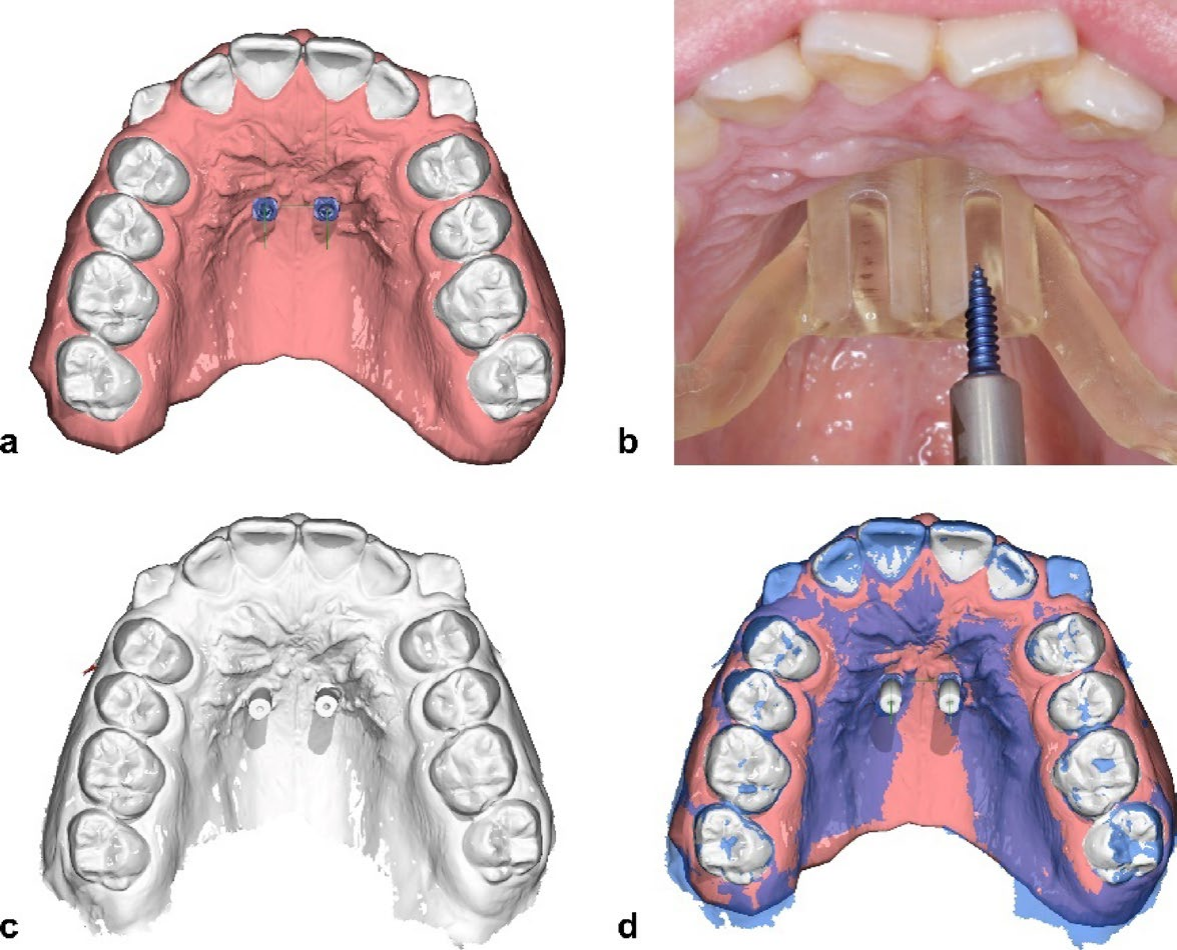
a, c, e: Skeletonized insertion guide design; b, d, f: Full arch insertion guide design; a, b: Top view; c, d: Back view; e, f: Side view. Displayed in Autodesk Meshmixer version 3.3.15 (San Francisco, CA, USA).

Two different guide designs were used in this study, which were designed using the OrthoApps module of the software:

- Group 1: Skeletonized design (Fig. 4a, c, e).
- Group 2: Full arch design (Fig. 4b, d, f).

The skeletonized design surgical guide (Group 1) is only supported on small occlusal surface areas and therefore allows better visibility during clinical TAD insertion, while the full arch design surgical guide (Group 2) provides a larger support surface for the guide on the occlusal surfaces of the teeth and the palatal mucosa, but thereby limits visibility (Fig. 4a-f)^14^. In both groups, vertical drilling depth was defined by the cylindrical, upward-facing surfaces of the guides, which matched the insertion tool to halt further advancement once the planned depth was reached. Therefore, a screwholder with stop was used (33-10903, PSM Medical GmbH, Gunningen, Germany). Patients were allocated to group 1 or group 2 by random selection using a lottery procedure by a blinded non-clinical scientist (UB). Specifically, all lots were prepared with an equal 50:50 distribution—half assigned to the skeletonized group and half to the full arch group. These lots were placed in a sealed lottery pot, accessible only to one designated person (UB). Before each draw, the lottery pot was shaken to ensure randomness. When a patient was recruited, one of two designated orthodontists (LH, HS) approached UB and, in his presence, drew a lot. The outcome of the draw determined whether the patient was allocated to the skeletonized group, or the conventional group and the result was documented in the study records.

The designed insertion guides were exported in STL format (Standard Tessellation Language) and 3D printed with a z-resolution of 50 μm using a stereolitography (SLA) 3D printer and biocompatible resin (Formlabs 3B + and Surgical Guide Resin, Formlabs Inc., Somerville, USA). Post-processing, including air-drying, washing and light-curing, was performed according to the manufacturer’s specifications.

#### Surgical TAD insertion and evaluation Procedure

**Fig. 5:**
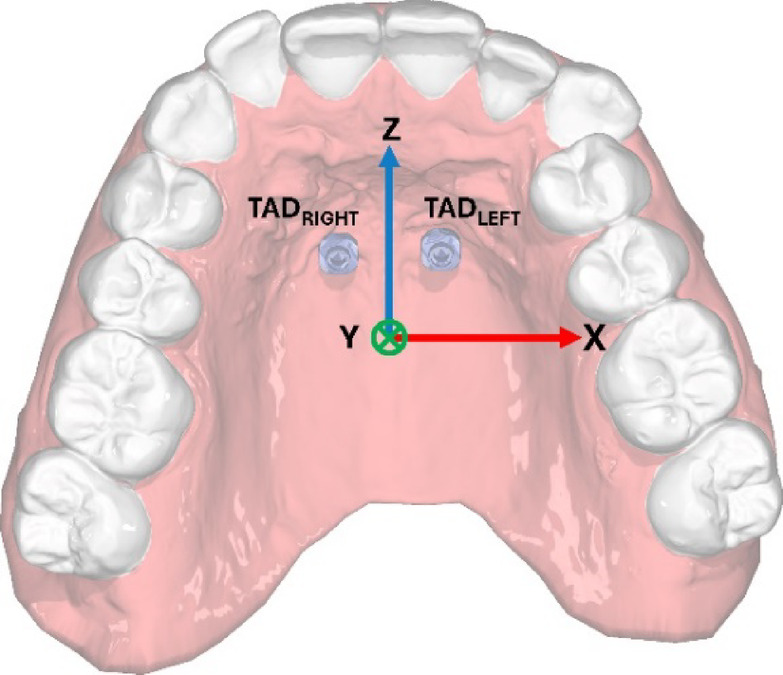
*a: Virtual planning of TAD positions; b: Intraoral image of surgical guide during TAD insertion with screw and screwholder with stop; c: Intraoral scan with scan-abutments; d: Matched virtual implant positions and scanned positions. Displayed in OnyxCeph version 3.2 (Image Instruments*,* Chemnitz*,* Germany*, https://onyxceph.eu*).*

After local anesthesia of the anterior palate, guided TAD insertion was performed with an electric screwdriver (implantmed, W&H Dentalwerk Bürmoss GmbH, Bürmoss, Austria) with an insertion moment of 30 Ncm and 30 rpm without pre-drilling. The left TAD was inserted first in the clinical protocol (Fig. 5b). After insertion, corresponding scan abutments were placed on the TADs and scanned with an intraoral scanner (Trios 3, 3shape A/S, Copenhagen, Denmark).

These scans were matched with the virtual planning model using a software function of the TADmatch module, applying an ICP (iterative closest point) algorithm (Fig. 5a, c, d). For the superimposition procedure, dental structures and the soft tissues of the anterior palate were considered relatively stable, given the short time frame between the planning and post-insertion scans, excluding the influence of aging or growth; furthermore, no orthodontic tooth movement was performed within this period. The anterior palate, particularly the region around the third palatal rugae, has been shown to provide a reliable reference for superimposition, even in growing patients^19^. After the superimposition, the x, y, z coordinates of the virtually planned TAD positions were exported. Second, the virtual TADs were registered to the position of the scanned TADs using coordinate transformation and supported by additional software functionality of the module. Then the x, y, z coordinates of the achieved TAD positions were exported.

#### Orthodontic treatment and follow-up period

After TAD insertion and intraoral scanning to assess accuracy, all TADs were loaded with orthodontic appliances and orthodontic force within three weeks after insertion. Depending on the clinical indication, mesialization and distalization appliances, as well as maxillary expanders were used for treatment. TAD success was assessed after a period of three months after clinical loading, removing the appliances temporarily for clinical evaluation. TAD success was defined as absence of inflammation signs and no mobility.

#### Calculation and statistical analysis

**Fig. 6:**
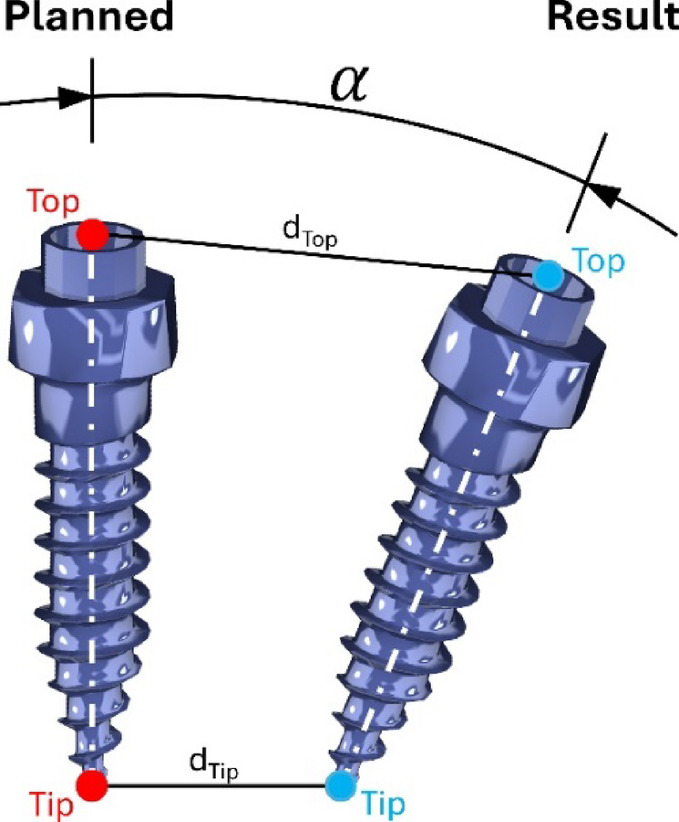
Evaluation measures between planned and resulting TAD: distance between top (d_Top_) and tip (d_Tip_) as well as angle (α) between the long axis of the TAD.

The measurement of differences between the planned and resulting TAD was calculated on basis of the 3D coordinates of the tip and top of the TADs (Fig. 6). The assessing researcher (LH) was blinded to the treatment groups and was only provided with the respective coordinates for analysis.

Calculations were performed in Python 3.11 using NumPy (version 1.26.3)^20^ and pandas (version 2.1.4)^21,22^. Three evaluation parameters were used: the distance between the top (d_Top_), the distance between the tip (d_Tip_) and the angle (*α*) between the long axis of the TADs (see Fig. 6).

The distance (d) was calculated as the length of the connecting vector ($\:\overrightarrow{\varvec{c}}$) either between the tip or the top of the planned and resulting TAD following the Euclidean norm in formula (1):

1 $$\:d=\parallel\overrightarrow{\varvec{c}}\parallel=\sqrt{{{(c}_{x})}^{2}+{{(c}_{y})}^{2}+{{(c}_{z})}^{2}\:}$$.

The angle (*α*) was based on the vectors defining the long axis between tip and top of the planned ($\:\overrightarrow{\varvec{u}}$) and resulting ($\:\overrightarrow{\varvec{v}}$) TAD using formula (2):

2 $$\:\alpha\:={arccos}(\frac{\overrightarrow{\varvec{u}}\:^\circ\:\:\overrightarrow{\varvec{v}}\:}{\parallel\overrightarrow{\varvec{u}}\parallel\cdot\parallel\overrightarrow{\varvec{v}}\parallel\:}\:)$$.

Statistical analysis was performed using IBM SPSS Statistics version 29 (Armonk, NY, USA). The metric data was tested for normal distribution using the Shapiro-Wilk test. Since several variables showed deviation from the assumption of normality, non-parametric tests were applied: significant differences were analyzed using the Kruskal-Wallis test, while the Wilcoxon test was used to check the dependent variables for significances. Further, the nominal data was tested for significant differences using the Pearson-Chi^2^-test. For all tests, a *p*-value < 0.05 was considered significant. A post-hoc power analysis was conducted using G*Power software (version 3.1.9.6 for macOS)^18^. The results are provided as Supplementary Table S1.

#### Error of the method

To quantify the error of the registration algorithm, two TADs in two cases were exemplarily reevaluated four times. Between the results a deviation error of ± 0 mm (or in degrees accordingly) was calculated.

## Conclusions

The full arch design showed significantly higher accuracy compared to the skeletonized design. During virtual planning of TAD positions, a safety distance should be considered to account for possible deviations using CAD-CAM surgical guides for TAD insertion in the anterior palate.

## Electronic supplementary material

Below is the link to the electronic supplementary material.

Supplementary Material 1

## Data Availability

Supplementary data will be shared on reasonable request to the corresponding author.
