# Supplementary material for: Accuracy of guided insertion of orthodontic temporary anchorage devices comparing two different 3D printed surgical guide designs: a randomized controlled trial
Source: Sci Rep. 2025 Aug 28;15:31749. doi: 10.1038/s41598-025-12116-1 (PMC12394681; doi:10.1038/s41598-025-12116-1)
Supplement: Supplementary file 1 — Supplementary Material 1 [file 41598_2025_12116_MOESM1_ESM.docx]

**Supplementary Table S1.** Post-hoc power analysis of the main findings. Post-hoc power analysis was done using G*Power (Version 3.1.9.6, Mac) with the following setting: t tests, ‘Means – Wilcoxon-Mann-Whitney test (two groups)’, Post-hoc, two-sided, α=0.05, N1=N2=40.

| **Variable** | **Full arch design** | |  | **Skeletonized design** | | ***p*-value** | **Effect size *d*** | **Power** |
| --- | --- | --- | --- | --- | --- | --- | --- | --- |
|  | **Mean** | **SD** |  | **Mean** | **SD** |  |  |  |
| Angle [°] | 5.34 | 4.86 |  | 6.44 | 3.36 | 0.041 | 0.263 | 0.206 |
| Distance tip [mm] | 1.42 | 1.29 |  | 2.00 | 1.01 | 0.005 | 0.500 | 0.579 |
| Distance tip in X [mm] | 0.49 | 0.40 |  | 0.57 | 0.53 | 0.838 | 0.170 | 0.114 |
| Distance tip in Y [mm] | 0.61 | 0.76 |  | 0.99 | 0.72 | 0.004 | 0.513 | 0.114 |
| Distance tip in Z [mm] | 1.00 | 1.15 |  | 1.47 | 0.89 | 0.004 | 0.457 | 0.505 |
| Distance top [mm] | 0.72 | 0.46 |  | 1.01 | 0.55 | 0.011 | 0.572 | 0.694 |
| Distance top in X [mm] | 0.32 | 0.29 |  | 0.37 | 0.29 | 0.339 | 0.172 | 0.115 |
| Distance top in Y [mm] | 0.35 | 0.35 |  | 0.61 | 0.50 | 0.008 | 0.602 | 0.739 |
| Distance top in Z [mm] | 0.40 | 0.38 |  | 0.57 | 0.42 | 0.033 | 0.424 | 0.448 |
